# Modes of North Atlantic Western boundary current variability at 36° N

**DOI:** 10.1038/s41598-023-45889-4

**Published:** 2023-10-31

**Authors:** Shun Mao, Ruoying He, Magdalena Andres

**Affiliations:** 1 North Carolina State University, Raleigh, NC USA; 2 Woods Hole Oceanographic Institution, Woods Hole, MA USA

**Keywords:** Ocean sciences, Physical oceanography

## Abstract

The surface-intensified, poleward-flowing Gulf Stream (GS) encounters the equatorward-flowing Deep Western Boundary Current (DWBC) at 36° N off Cape Hatteras. In this study, daily output from a data-assimilative, high-resolution (800 m), regional ocean reanalysis was examined to quantify variability in the velocity structure of the GS and DWBC during 2017–2018. The validity of this reanalysis was confirmed with independent observations of ocean velocity and density that demonstrate a high level of realism in the model’s representation of the regional circulation. The model’s daily velocity time series across a transect off Cape Hatteras was examined using rotated Empirical Orthogonal Function analysis, and analysis suggests three leading modes that characterize the variability of the western boundary currents throughout the water column. The first mode, related to meandering of the GS current, accounts for 55.3% of the variance, followed by a “wind-forced mode”, which accounts for 12.5% of the variance. The third mode, influenced by the DWBC and upper-ocean eddies, accounts for 7.1% of the variance.

## Introduction

The Gulf Stream (GS) is a surface-intensified western boundary current in the North Atlantic Ocean that undergoes a transition near Cape Hatteras where it changes from a flow “attached” to the offshore edge of the continental shelf to a free jet in the open^1,2^. As the GS approaches Cape Hatteras from the south along the South Atlantic Bight, the flow is primarily along the isobaths of the steep continental slope with small amplitude meanders^3–5^. After it separates from the continental shelf near Cape Hatteras (at 36°N) and flows across isobaths into the deeper Atlantic Ocean, the GS exhibits large amplitude meanders^6^ that can grow to form warm and cold core rings.

In this transition region near Cape Hatteras, the Deep Western Boundary Current (DWBC), which flows towards the equator, encounters the poleward-flowing GS. The interaction between the GS and DWBC has been observed in earlier studies^7–15^. Different conclusions about the interaction of the GS and DWBC near Cape Hatteras have been reached due in part to limitations in spatial and temporal resolution and duration of in situ observations. Some early studies^9^ used hydrographic data to conclude that the GS entrains a shallow layer of Labrador Sea Water (LSW), with only the onshore-most part of this intermediate layer continuing to flow equatorward past Cape Hatteras along the boundary. However, the Boundary Current Experiment (BOUNCE) program (1994–1995) used Lagrangian floats to show that all Upper Labrador Sea Water (ULSW) is entrained by the GS and none of it flows south past Cape Hatteras^12,13^. More recent studies have provided contrasting results using different observational techniques. For instance, 25 years of chlorofluorocarbon observations^14^ inferred that young ULSW flows along the deep boundary and is little diluted by older interior water between 42°N and 26°N. Six GS cross-slope velocity fields^15^ indicated a continuous DWBC flow beneath the GS near Cape Hatteras, including the deepest part of the ULSW and at least the top of the Classic Labrador Sea Water (CLSW).

The interaction between the GS and DWBC near Cape Hatteras may be influenced by GS meanders and local wind forcing. Moored current meter data^16^ from the continental shelf and slope near Cape Hatteras suggested that the variability of along-shelf transport of the GS is primarily driven by the wind and is highly correlated with sea level fluctuations at the coast. Ocean gliders observed that GS transport was reduced by 40% for approximately two weeks following the passages of Hurricanes José and Maria^17^. Atmospheric conditions near Cape Hatteras were classified into warm season (May to mid-September) and cool season (mid-September through April)^18^. During the cool season, energetic extratropical cyclones induce strong wind stress that accounts for over 40% of the total current variability over the continental shelf near the Cape Hatteras region^18^.

The open questions from these earlier studies, and the scarcity of direct measurements of the deep ocean near Cape Hatteras, motivated us to study the variabilities of the western boundary currents (GS and DWBC) at 36°N off Cape Hatteras using a high-resolution, data assimilative ocean model. This model assimilates observations from multiple platforms, including satellite observations and observations from an array deployed as part of the National Science Foundation-funded Processes Driving Exchange at Cape Hatteras (PEACH) project^15,19–22^. Section "Data" describes the model configuration and validation, while Section "Analyses" shows the model results and the temporal and spatial variability of the boundary current flowing through cross-shelf sections near Cape Hatteras using rotated Empirical Orthogonal Functions (EOFs). Finally, Section "Summary" presents a summary of our findings.

## Data

### Regional Ocean Reanalysis

**Figure 1:**
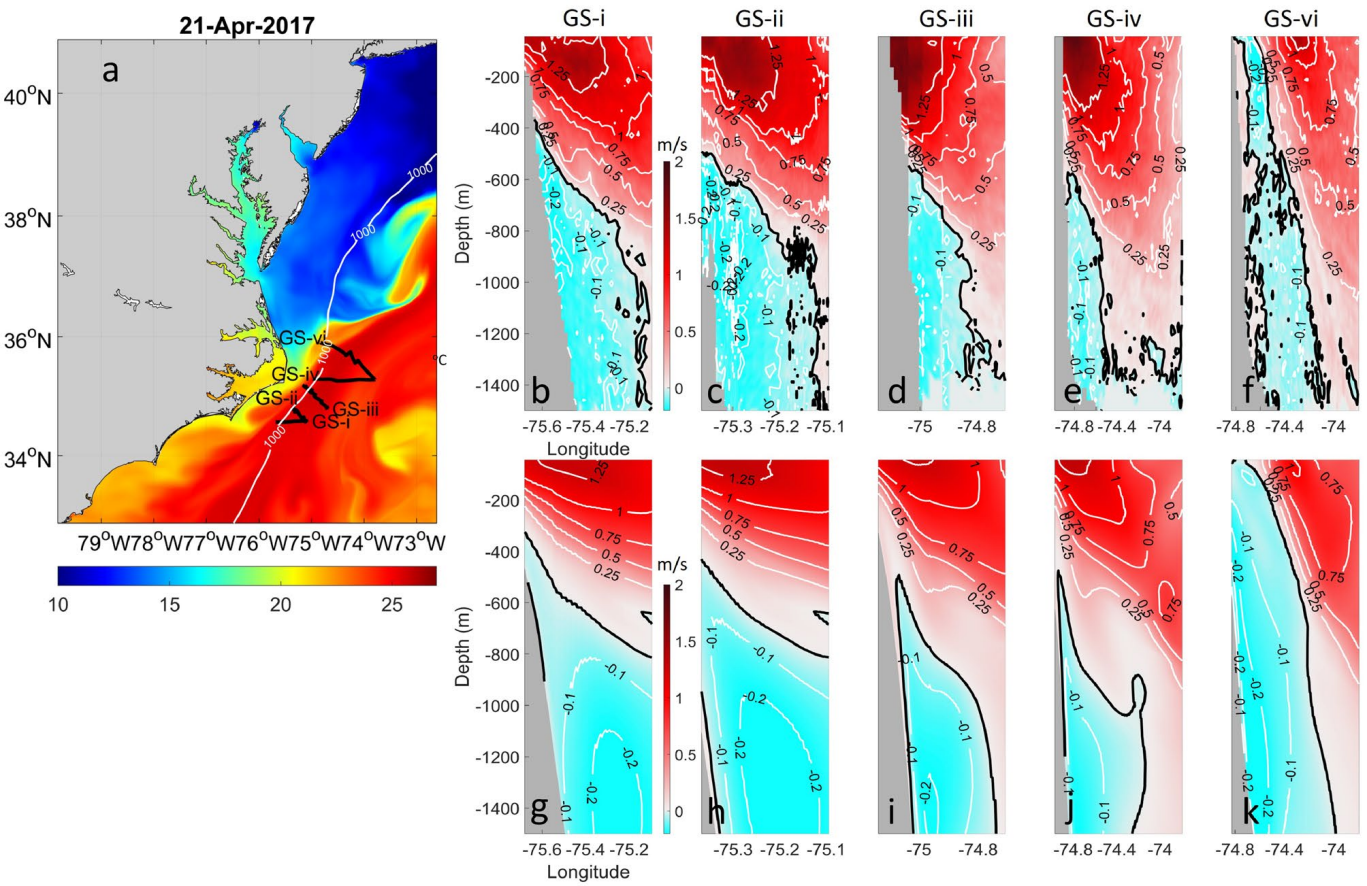
(**a**) Color shading represents sea surface temperature from our data assimilative model on April 21, 2017. The five cross-shelf transects (GS i-v) are depicted as black solid lines, along with the 1000-m isobath shown as a white solid line. In (**b**) GS-i, (**c**) GS-ii, (**d**) GS-iii, (**e**) GS-iv, and (**f**) GS-vi, we display the observed upper 1500-m cross-track velocity fields along these five transects. We used the orientation of the 1000-m isobath, which sharply curves around the Cape, to define “equatorward” flow at each section. Specifically, at GS-i, GS-ii, GS-iii, GS-iv, "equatorward" flow (negative velocity) is defined as current moving toward 210° from true north, while positive flow is defined as current moving toward 30° from true north. At GS-vi, "equatorward" flow (negative velocity) is defined as current moving toward 180° from true north, while positive velocity is defined as current moving toward true north (0°). The black solid line in each sectional contour map indicates 0 m/s. Velocity fields are contoured at 0.25 m/s for positive values and 0.10 m/s for negative values. The maximum depth of the velocity profiles from the Ocean Surveyor 38kHz ADCP is about 1500 m. Corresponding data-assimilative modeled velocity fields along the same five transects are shown in panels (**g**–**k**).

The ocean reanalysis utilized in this study combines a high-resolution Regional Ocean Modeling System (ROMS)^23,24^ with an ensemble data assimilation approach to incorporate available remote and in situ ocean observations^25^. The model domain covers the South Atlantic Bight (SAB), Cape Hatteras, the Middle Atlantic Bight (MAB), and the adjacent coastal ocean (Fig. 1a). The model features a horizontal resolution of 800 m and 50 terrain-following vertical layers with higher resolution near the surface and bottom boundary layers. The atmospheric forcing data are from the European Centre for Medium-Range Weather Forecasts (ECMWF) interim products, while global Hybrid Coordinate Ocean Model (HYCOM) data are used for initial and boundary conditions. Downscaling from HYCOM to ROMS involves translating data between two different coordinate systems. This is done by first applying a horizontal interpolation. Then HYCOM’s data are vertically interpolated onto ROMS' sigma-coordinates. Tidal forcing is based on 13 major tidal constituents from the Finite Element Solution (FES) 2014 tide model^26^, and river forcing is based on 22 estuary rivers in the model domain extracted from the National Water Model. The model simulation period spans January 2017 to December 2018 when extensive in situ observations were available from the PEACH project. The model assimilates along-track sea level anomaly data from satellites active during the period comprising Jason-2, Jason-3, CryoSat-2, SARAL-AltiKa, Haiyang-2A, and Sentinel-3. It also incorporates daily multi-sensor combined sea surface temperature data from the Advanced Very High Resolution Radiometer (AVHRR), archived by NOAA CoastWatch. Furthermore, the model assimilates PEACH observations, including high-frequency radar, temperature and salinity profiles from glider surveys and moorings, and data from National Data Buoy Center (NDBC) buoys located near Cape Hatteras. The model time step is 60 s, and the output frequency is daily.

### Independent validation of the reanalysis

During the PEACH deployment cruise in April 2017 aboard the RV *Neil Armstrong*, cruise AR-15, six velocity sections across the GS were collected using the shipboard acoustic Doppler current profilers (ADCPs)^15^. These data are not assimilated into the model and serve here as independent observations for assessment of the ocean model performance. The northeastern section, GS-v, is excluded from the comparison as it was interrupted for full-water-column conductivity-temperature-depth (CTD) casts and covered nearly the same section as crossing GS-vi, which was not interrupted for CTD casts. It took 3.3, 4.75 and 8 h to complete ADCP crossing GS-i, GS-iv and GS-vi, respectively. The crossings of GS-ii and GS-iii required 10.4 and 16.2 h due to interruptions for CTD casts, as reported by Andres et al. (2018). Daily mean velocity sections from the model that are contemporaneous with each shipboard ADCP crossing are utilized for independent comparison and evaluation of model performance. Although tides are averaged out in the model daily output, the ADCP velocity data for the Gulf Stream sections are not detided since the barotropic tidal velocities in the Cape Hatteras region are generally weak^15^. Their contribution to velocities in this area is less than 0.1 cm/s.

The shipboard ADCP velocity sections, obtained over a window of ± 3 days around April 21, show an equatorward-flowing DWBC beneath the surface-intensified, poleward-flowing GS (Fig. 1b–f). This DWBC flow generally follows the 1000 m isobath. At GS-vi, where the 1000-m isobath is nearly meridional, the equatorward flow is directed southward (toward 180°). At crossings GS-i through GS-iv, where the isobaths are roughly toward 30°, the equatorward flow is directed toward 210°.

To estimate the total GS transport measured at each section, the ADCP velocity profiles are rotated to obtain downstream (v_rot, poleward positive) and cross-stream (u_rot, onshore positive) components, with directions based on the maximum surface velocity vector observed at each section^15^. The transport for each section is then obtained by integrating over the area of all positive v_rot. The observed GS transport across the sections increases downstream from 27.8 Sv at GS-i to 58.6 Sv at GS-iv. The horizontal endpoints chosen to define the GS can strongly influence GS transport calculations. The GS transports calculated here from the ADCP GS crossings are likely underestimates since the shipboard transects did not cover the whole cross-sectional width of the GS (i.e., they did not reach to a 0 m/s isotach marking the GS offshore edge).

These in situ observations are used to help assess the model’s representation of the DWBC and the GS. For the most direct comparison, daily averaged model output is extracted along the same five transects with the same endpoints as the ADCP sections and on the same day as each ADCP section (Fig. 1g–k). To run ROMS, it was necessary to smooth the bathymetry to minimize the pressure gradient errors associated with the sigma-coordinate ocean model, thus there are some discrepancies in bathymetry between the observations and the model near the shelfbreak area. Nevertheless, model results show a persistent equatorward-flowing DWBC beneath the onshore edge of the poleward-flowing GS that is consistent with the observations. Moreover, both modeled and observed DWBC velocity fields in Fig. 1 reveal that the equatorward DWBC is weaker at section iv than at section vi, but it accelerates as it flows from GS-iv to GS-ii where the isobaths are more tightly spaced.

The model results are also consistent with the observations of the GS. Using the same method to calculate the modeled GS transport (i.e., daily snapshots and the same section endpoints for the velocity integrations), the comparison (not shown) indicates that both model and observations have an increase in GS transport downstream (from south to north) by more than a factor of two, with model GS transport increasing from 26.3 Sv at GS-i to 65.7 Sv at GS-iv. The local intensification of the GS transport near Cape Hatteras is consistently observed in both the model and shipboard ADCP observations.

However, limited by the vertical resolution of the model in the interior away from the boundaries, the model snapshots tend to overestimate the transport in locations where the GS’s maximum velocity core is further offshore with greater water depth. For instance, at transect GS-vi the 0.75 m/s contour in the model extends further offshore and deeper than it does in the corresponding observed section, as shown in Fig. 1f,k. From GS-i to GS-iv, the total number of vertical layers in the model remains the same but the water depth increases, so that the model layers become more spread out in the water column. Overestimation of GS transport at GS-iv by the model compared to the observed ADCP sections is primarily due to the model’s vertical resolution within the GS core.

**Figure 2:**
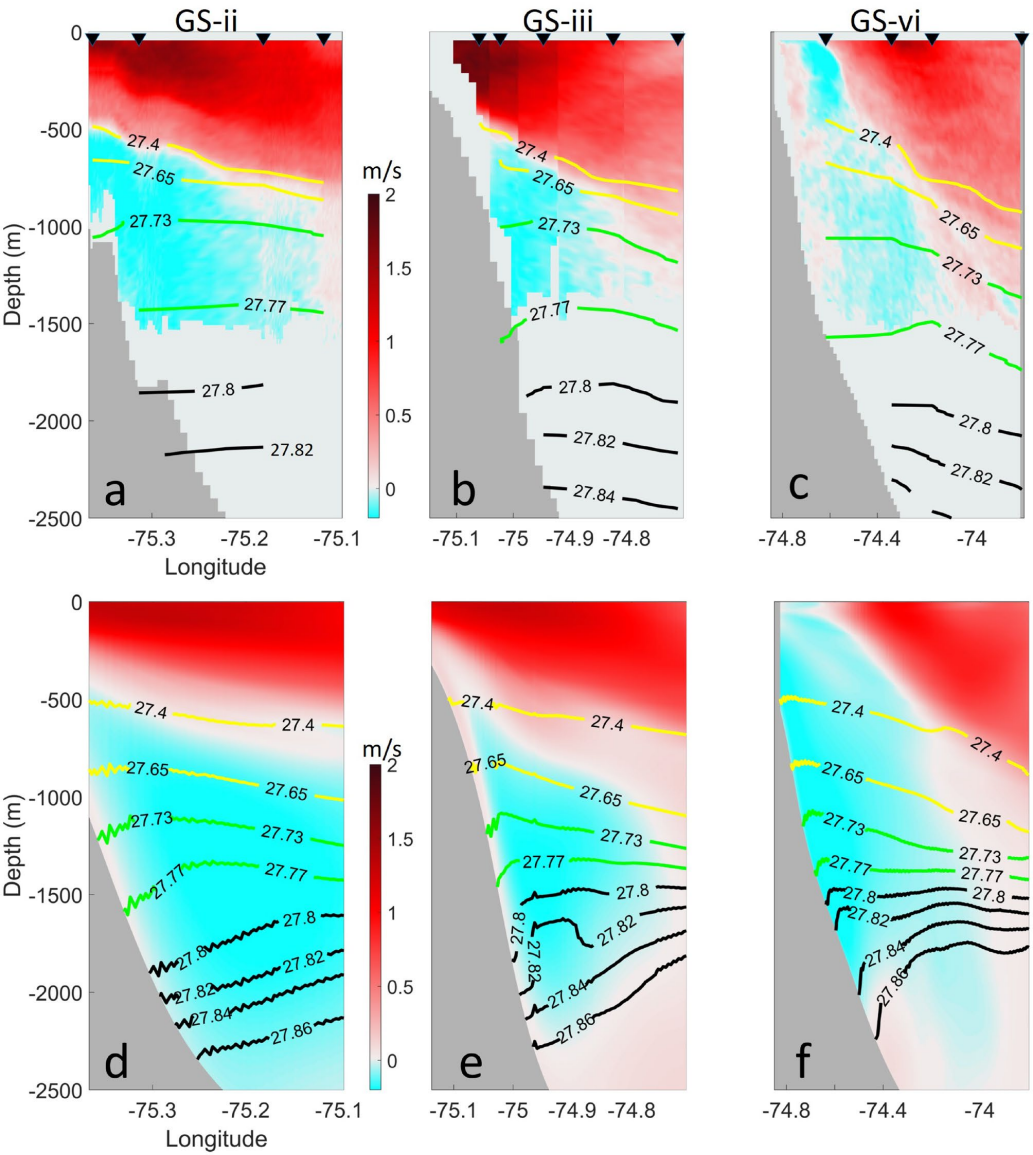
Comparisons of potential density fields of the upper 2500 m of the water column between in-situ observations and data-assimilative model results along three ship-track transects (GS-ii, GS-iii, and GS-vi). The upper panels show the potential density contours from observations, overlaid with observed velocity fields. Black triangles in (**a**–**c**) indicate the locations of CTD casts. The lower panels show the potential density contours from the model, overlaid with modeled velocity fields.

The subsurface equatorward flow near Cape Hatteras measured by these shipboard ADCP sections comprises primarily the upper part of the DWBC^15^, based on neutral density using water mass definitions^27^, and may also contain a thin layer of Labrador Slope Water (LSLW), a water mass with potential density (${\upsigma }_{0}$) ranging between $27.4\mathrm{ kg}/{\mathrm{m}}^{3}$ and $27.65\mathrm{ kg}/{\mathrm{m}}^{3}$ formed from the Labrador Current on the Labrador Shelf^28^. In Fig. 2, we compare potential density referenced to the surface (${\sigma }_{0})$ between the observations and model results. During the deployment cruise AR-15, profiles of temperature and salinity were measured with CTD casts at several stations along GS-ii, GS-iii and GS-vi, and the observed potential densities in the upper 2500 m of the water column are contoured in Fig. 2a–c. The model-simulated potential density is shown in Fig. 2d–f. The water mass with a potential density (${\upsigma }_{0}$) ranging between $27.4\mathrm{ kg}/{\mathrm{m}}^{3}$ and $27.73\mathrm{ kg}/{\mathrm{m}}^{3}$ was classified as the lightest Labrador Sea Water^9,27^, though this may also include a layer of LSLW^28^. The water mass with a potential density falling within the range of $27.73\mathrm{ kg}/{\mathrm{m}}^{3}$ to $27.77\mathrm{ kg}/{\mathrm{m}}^{3}$ was defined as intermediate Labrador Sea Water^9^. Both the observations and model results indicate continuous presence of the lightest Labrador Sea Water and intermediate Labrador Sea Water between 500 and 1500 m at GS-ii to GS-iv. Additionally, denser Labrador Sea Water (${27.8\mathrm{ kg}/{\mathrm{m}}^{3}<\upsigma }_{0}<27.82\mathrm{ kg}/{\mathrm{m}}^{3})$ is present continuously from the northernmost transect (GS-vi) to the southern transect (GS-ii). The denser Labrador Sea Water is observed near 2000 m depth, while in the model, it is at about 1500 m. This difference is likely caused by the model’s relatively coarse vertical resolution in the middle of the water column.

In summary, these independent velocity and water mass comparisons for several “snapshots” indicate the model has good skill in generating the along-stream and cross-stream spatial structures where the GS and DWBC cross. In addition to these snapshot comparisons, the model is also validated by comparison of the model output with timeseries of observation-based thermocline depth inferred from current and pressure sensor equipped inverted echo sounders (CPIESs) deployed for 18-months as part of the PEACH program (Andres 2021, Fig. 6). These comparisons with the model-derived thermocline depths extracted at the CPIES locations have a correlation coefficient r = 0.7(figure not shown). Because the CPIESs data are not assimilated into the model, this provides an additional independent measure of the model skill. This, together with the velocity sections, gives us confidence to use the model output to further examine the spatial structure and the temporal variability at the crossover.

## Analyses

### Mean state of vertical profiles of western boundary currents near Cape Hatteras

**Figure 3:**
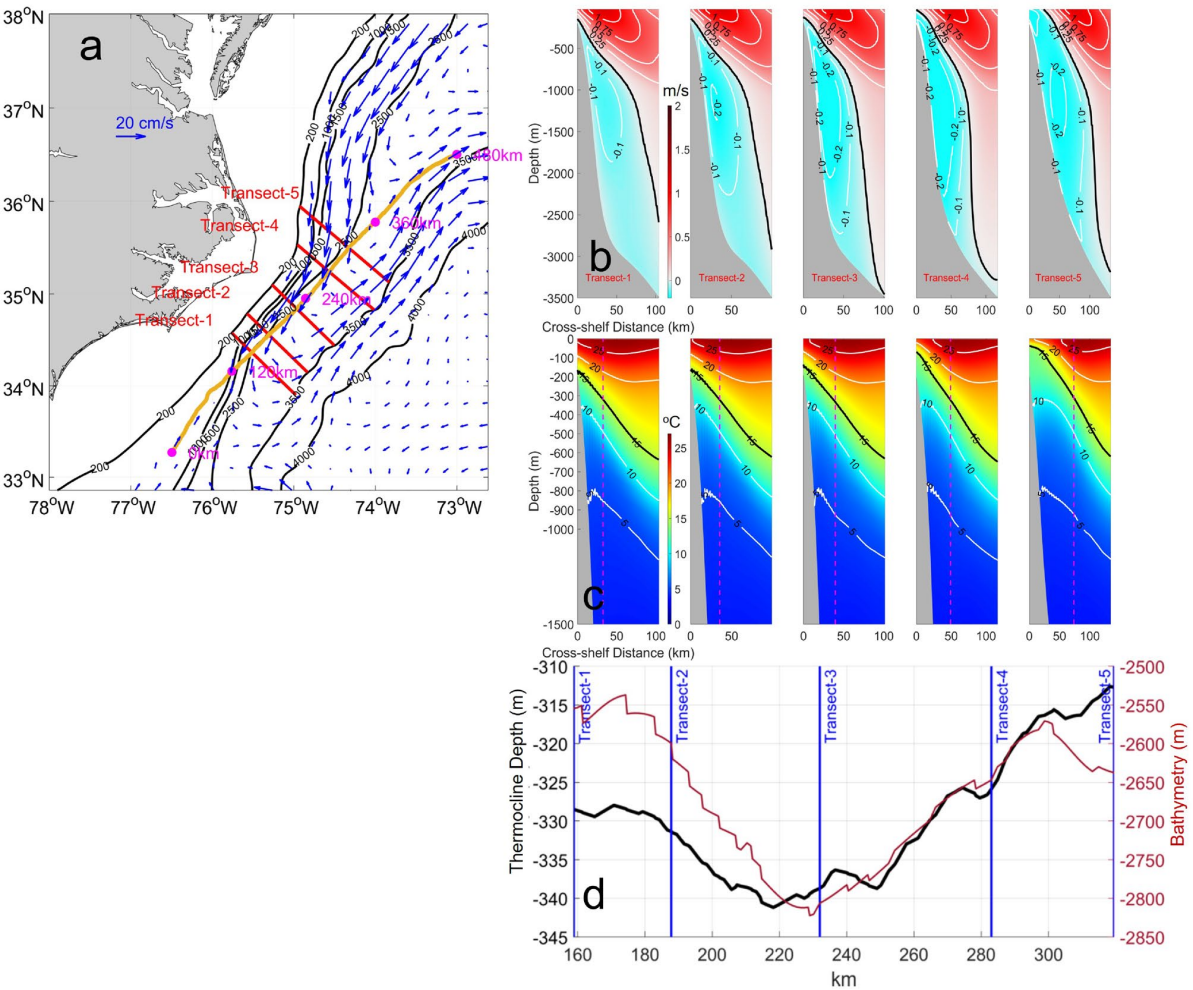
(**a**) The blue vectors represent the velocity field averaged vertically within the Upper Labrador Sea Water (ULSW) layer. The ULSW layer is characterized by specific potential density boundaries; the upper boundary is defined by a potential density (${\sigma }_{0}$) of 27.4 kg/m^3^, while the lower boundary is determined by a potential density (${\sigma }_{0}$) of 27.77 km/m^3^. Black lines mark the 200, 1000, 1500, 2500, 3500 and 4000 m isobaths. The orange line denotes the two-year mean Gulf Stream (GS) path, defined by the surface maximum velocity from the model. Five cross-shelf GS model transects are plotted in magenta lines. (**b**) Two-year mean poleward velocities (perpendicular to each transect), contoured at 0.25 m/s for positive values and 0.10 m/s for negative values. (**c**) Two-year mean temperature structures for the upper 1500 m (transects 1–5, from left to right). Black lines highlight the 15 °C isotherm. The magenta lines show the locations of the two-year mean position of the GS center along the transect. (**d**) Two-year mean 15° isotherm depth along the mean GS path (orange line in **a**) is shown by the black line. The ocean floor depth along the mean GS path is shown by the red line.

During cruise AR-15, the ship transects did not span the entire width of the GS in the cross-stream direction. Therefore, five new cross-stream transects spanning the 200 m isobath to the 3500 m isobath (or to the 4500 m isobath for mean transport calculations, see below) are defined in the model to better capture the full width of the GS. These new transects, numbered 1 through 5 from south to north, are each perpendicular to the two-year mean (modeled) GS path near Cape Hatteras. The maximum *gradient* of the two-year mean sea surface height (SSH) is used to identify the model’s mean GS path, which is represented by an orange solid line in Fig. 3a. This method more accurately captures the location of the GS core near Cape Hatteras than does the method^29^ which relies on a constant SSH contour as a proxy for GS path^30^. The vertically averaged deep velocity field (shown by blue vectors in Fig. 3a), is calculated for the layer that falls within the potential density range of ${27.4 \mathrm{kg}/{\mathrm{m}}^{3}<\sigma }_{0}<27.77 \mathrm{kg}/{\mathrm{m}}^{3}$. This suggests that the ULSW (and possibly part of the LSLW) that flows equatorward towards Cape Hatteras between the 1000-m and 2500-m isobaths is subsequently entrained by the deep GS south of Cape Hatteras, near 34.5 N°. The deep GS flows northeastward between the 3000-m and 4000-m isobaths and is located further offshore compared to the upper GS (consistent with observations^31^).

To distinguish between the "poleward" and "equatorward" directions mentioned previously, the two-year mean velocities in the model were rotated based on the local orientation of the mean GS path to obtain cross-transect normal $\overline{{v }_{nor}}$ and cross-slope $\overline{{u }_{tan}}$ components (Fig. 3b). This rotation method is consistent for both the GS and DWBC, allowing for the estimation of their transports in the same coordinate system. The equatorward-flowing DWBC is clearly identified beneath the onshore edge of the poleward-flowing GS. The core of the GS (identified by the 1 m/s contour) is shifted further offshore by about 30 km from south (transect 1) to north (transect 5) where the GS separates from the continental margin. The location of the surface temperature maximum regions (Fig. 3c) aligns well with the two-year mean positions of the GS, which are identified by the maximum gradient of SSH.

The 2-year mean DWBC transport is estimated by integrating over the area of negative $\overline{{v }_{nor}}$ in each transect. Where the DWBC approaches Cape Hatteras, its transport gradually declines from 22.3 Sv, at transect 5 to 10.6 Sv at transect 1. To estimate the mean transport of the GS, transects are extended offshore to the 4500-m isobath to reach the GS's offshore edge (at the 0 m/s isotach), and after integrating over all the area of positive $\overline{{v }_{nor}}$ , it is found that GS mean transport remains relatively constant in the downstream direction. Specifically, GS mean transport is 67.3 Sv, 70.4 Sv, 75.2 Sv, 73.8 Sv, and 73.5 Sv at transects 1, 2, 3, 4 and 5, respectively.

Thermocline slope is another useful variable for characterizing the vertical structure of western boundary currents. The 15 °C isotherm (black lines in Fig. 3c) is an indicator of the GS thermocline, and the position of the 15 °C isotherm at 200 m depth has been widely used to track GS position^32,33^. The mean slope of the 15 °C isotherm across the GS gradually increases from south (transect 1) to north (transect 5), supporting previous studies’ findings^34,35^ that GS speed and poleward transport gradually increase downstream not only over the broad region between the Straits of Florida and the New England Seamount Chain, but also locally where the GS separates from the continental shelf and flows into the deep ocean (67.3 Sv at transect 1 and 73.5 Sv at transect 5).

Further analysis reveals that the thermocline depth deepens by 10 m over a 73 km distance from transect 1 to 3, then rises approximately 30 m over a 100 km distance from transect 3 to a midpoint between transects 4 and 5 (Fig. 3d). These spatial (along-stream) variations in thermocline depth are correlated with changes in ocean bathymetry, as the equatorward-flowing DWBC strives to maintain a consistent layer thickness to conserve its potential vorticity^36^. However, the maintenance of a constant layer thickness in the DWBC does not completely account for the observed variations in thermocline depth. Along the GS core there is an order of magnitude greater change in bottom depth than in modeled thermocline depth: a 200 to 300-m change in ocean floor depth is accompanied by only a 10 to 30 m variation in thermocline depth. Future studies may consider other factors impacting thermocline depth variations and explore the relationship between the different components of the DWBC^27^ and how changes in ocean bathymetry affect each DWBC layer.

### Rotated EOF analysis

Empirical Orthogonal Functions (EOFs) are used to examine the temporal and spatial variability of the modeled cross-shelf velocity fields near Cape Hatteras. This analysis is performed on the five cross-stream transects shown in Fig. 3a. Prior to the EOF analysis, two-year average values are subtracted from the total velocity fields. The two-year average is obtained by summing the velocity values over all discrete times ${t}_{j}$ in the two-year period (from $j=1 to N$) and then dividing by $N$, where $N$ is the total number (730) of days in those two years.

1 $${{\varvec{u}}}^{\prime}\left(x,t\right)={\varvec{u}}\left(x,t\right)-\frac{1}{N}\sum_{j=1}^{N}u\left(x,{t}_{j}\right)$$

**Figure 4:**
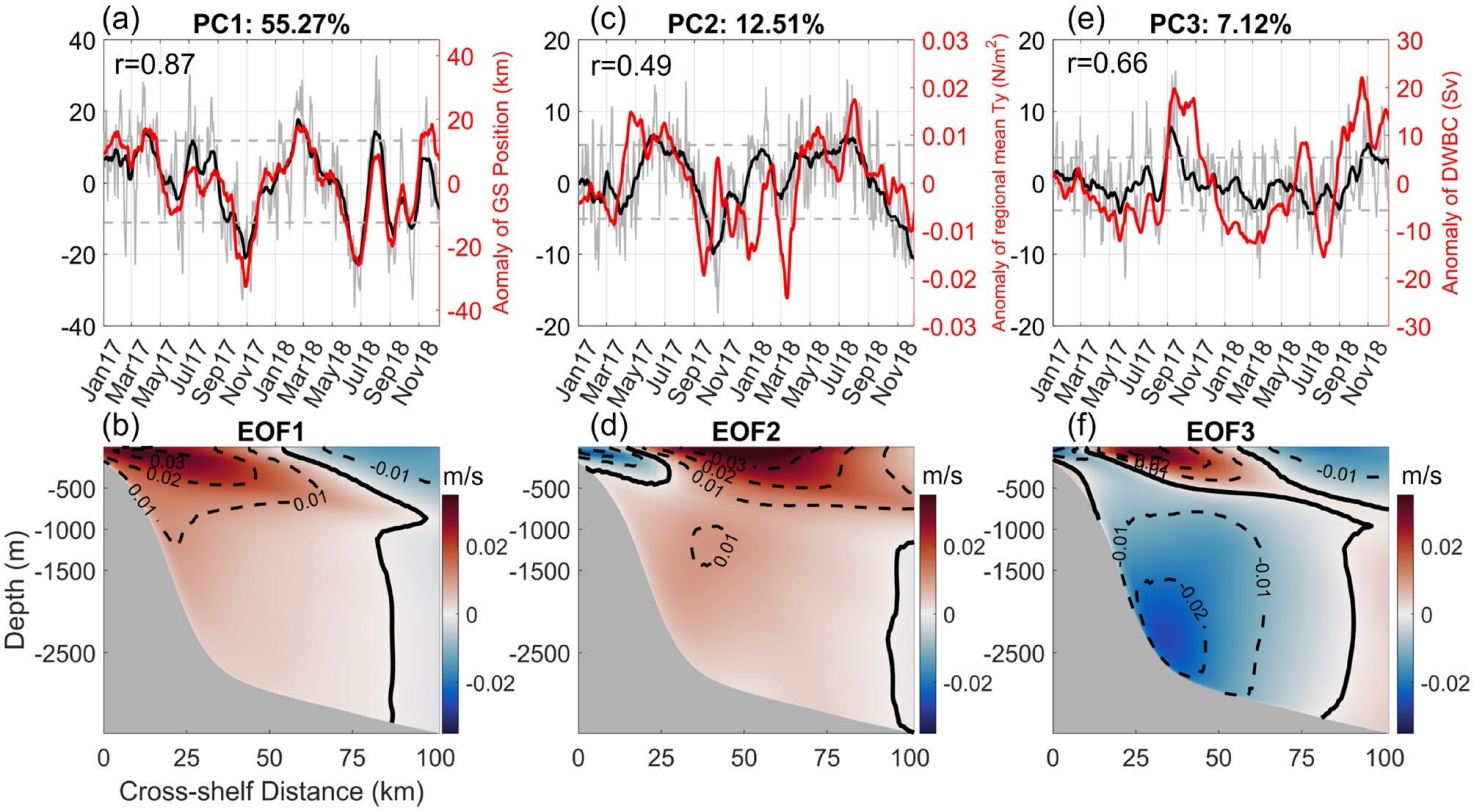
The results of first three rotated Empirical Orthogonal Function (EOF) modes at transect 3 (see Fig. 3a). The upper panel presents three principal components (PCs) in grey lines. The 30-day running mean PCs are shown with black lines. Red lines show (**a**) anomaly of GS position, (**c**) anomaly of regional mean of meridional wind stress T_y_, and (**e**) anomaly of transport of the DWBC at transect 3 in PC1, PC2, and PC3, respectively. Positive (negative) anomaly of GS position indicates an onshore (offshore) meander. Correlation coefficients (r) between time series of anomalies and corresponding PCs are given in the upper left corner of the upper panel figures, with a 95% confidence level. The lower panel shows the corresponding velocity variance of EOF1, EOF2, and EOF3.

Decomposing velocity fields into EOFs, ${{\varvec{u}}}^{\prime}\left(x,t\right)$ can be represented by:

2 $${{\varvec{u}}}^{\prime}\left(x,t\right)=\frac{1}{N}\sum_{j=1}^{N}{a}_{n}(t){F}_{n} (x)$$

where $x$ represents a position vector, indicating the horizontal and vertical locations within the cross-stream transect. For each EOF mode, ${a}_{n}$ is a temporal evolution function and ${F}_{n}$ is the spatial eigenfunction. Because of its orthogonality constraint, EOF analysis may produce unphysical modes. Previous studies have shown that the drawbacks of EOF analysis can be reduced by rotated EOF (REOF) analysis^37^. This REOF approach is carried out by performing a varimax rotation^38,39^, which helps reduce the variances in the projection of the data, thereby putting the EOF basis closer to the actual data variability and increasing physical interpretability. In the process of REOF, we retain 10 leading EOF modes. All five new cross-stream transects show similar REOF results, so we focus here on transect 3. Figure 4 presents the principal components (PCs) and corresponding velocity variances of the first three leading modes along transect 3, which is located just offshore of Cape Hatteras. As described below, these three modes represent a GS position (meander) mode, a wind-forced mode and a mode that combines the influence of DWBC variability and arrival of offshore eddies at the transect.

The first EOF mode captures 55.3% of the modeled velocity variance. The spatial pattern associated with this mode, shown in Fig. 4b, illustrates positive velocity anomaly in the nearshore region and negative velocity anomaly in the offshore region, consistent with onshore/offshore movement in the position of the GS. In order to verify that EOF mode-1 represents a “GS position mode” and to help diagnose the physical driver(s) of variability in PC1, PC1 is compared directly to the model’s time-varying GS position, defined here by the location of the maximum surface velocity along the transect to create a model index of GS offshore distance. The 30-day running mean of PC1 (depicted by black line in Fig. 4a) demonstrates a high correlation with the index of GS position anomaly (depicted by red line in Fig. 4b), with a correlation coefficient of 0.87. Both the PC1 and index of GS position anomaly reveal that the GS meandered offshore during October 2017 and July 2018 and had an onshore displacement in January–February 2018 near Cape Hatteras.

**Figure 5:**
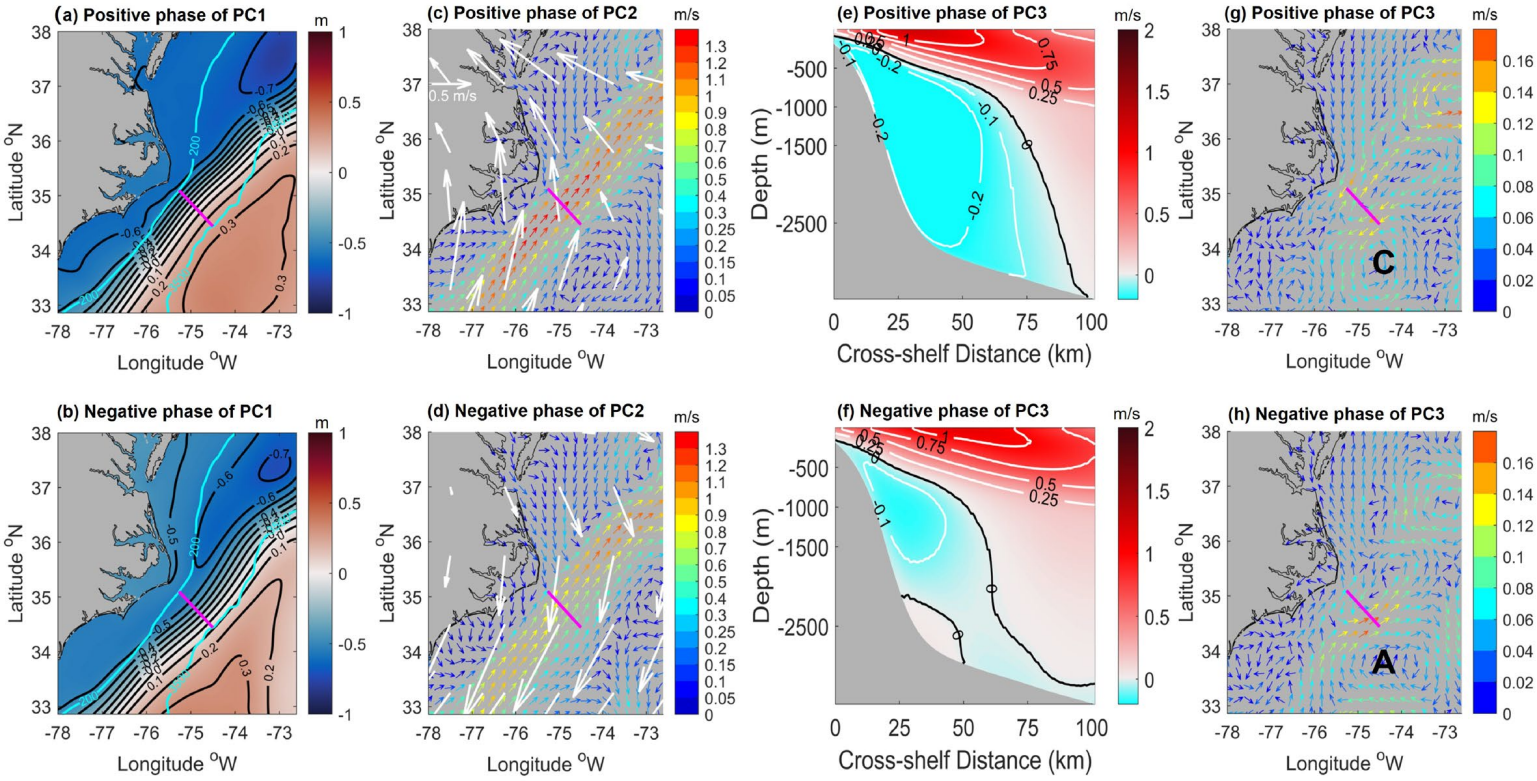
Positive and negative composite mean maps. The positive composite is averaged when daily PC is over its 80th percentile and the negative composite is averaged when daily PC is below its 20th percentile. (**a**) and (**b**) show composite mean sea surface height fields for the positive phase and negative phase of PC1, respectively. Cyan solid lines indicate the 200 m and 3500 m isobaths. Magenta lines show the location of transect 3. (**c**) and (**d**) show the positive and negative composite mean sea surface velocity (SSV) fields of PC2. Vector color denotes the magnitude of velocity. Corresponding wind anomaly vectors are overlaid in white vectors. (**e**) and (**f**) show the positive and negative composite mean velocity fields normal to transect 3 from PC3. (**g**) and (**h**) show positive and negative composite mean SSV anomaly based on EOF3. The capital letter 'C' in figure (**g**) indicates the location of a cyclonic eddy, while the letter 'A' in figure (**h**) indicates the location of an anti-cyclonic eddy near the Cape Hatteras transect.

To provide spatial context for the variability of this mode at this transect, composite SSH maps are created to define a positive phase by averaging SSH over periods when daily PC1 is over its 80th percentile and the negative phase by averaging over the periods when daily PC1 is below its 20th percentile. The positive and negative phases of the composite mean of SSH fields (Fig. 5a,b) demonstrate that during the positive (negative) PC1 phase, the GS mean path is located farther onshore (offshore). Notably, the shape of the − 0.6 m contour in Fig. 5b indicates a stronger southward flow along the outer shelf/upper slope when the GS is situated further offshore. This suggests that the Slope Sea gyre can extend further south and even reach north of Cape Hatteras under the negative phase of PC1, when the GS meanders further offshore. This comparison of SSH composites suggests that GS meandering is linked to the coastal circulation near Cape Hatteras and to the Slope Jet and the southwestern extent of the Slope Sea gyre offshore of the southernmost MAB^40,41^.

The second EOF mode, identified here as the “wind-forced mode”, accounts for 12.5% of the velocity variance across transect 3. EOF2 (depicted in Fig. 4d) displays a marked pattern of positive velocity anomaly in the GS core region and a weak negative velocity anomaly onshore of this over the upper continental slope and outer shelf. The domain-averaged meridional wind stress is used to investigate the influence of wind forcing, and Fig. 4c illustrates the 30-day running mean of PC2 (black line) and the anomaly of domain-averaged meridional wind stress (red line). PC2 is positively correlated with meridional wind stress (r = 0.49), with positive PC2 associated with upwelling-favorable winds during the summer season (June to September) and negative PC2 associated with downwelling-favorable wind conditions during the winter season (November through January). A Nor’easter event in March 2018 caused an unusual signal (depicted by the red line in Fig. 4c). Bane et al. associated the occurrence of strong Extratropical Cyclones (ETCs) off Cape Hatteras in March 2018 with the anomalously southern positioning of the atmospheric jet stream.

Composite sea surface velocity maps, overlaid with wind vector anomalies, are used to examine the physical mechanism that drives the second mode of variability. Similar to the composite SSH maps used to examine EOF1, a positive phase composite average is defined for EOF2 by averaging the sea surface velocities and the wind anomalies when daily PC2 exceeds its 80th percentile and the negative phase composite average is defined when daily PC2 falls below its 20th percentile. During the negative phase, which is the period of downwelling-favorable winds (Fig. 5d), the poleward-flowing GS slows down. During the positive phase, under the influence of upwelling-favorable winds (Fig. 5c), the GS’s velocity increases. The comparison of sea surface velocity composites provides a clear demonstration of how the variability in the GS region's velocity can be attributed to wind forcing.

The third EOF mode accounts for 7.1% of the velocity variance and is characterized by two distinct vertical features (Fig. 4f). One feature is the DWBC signal, while the other is a dipole in the upper 500-m water column. Unlike the first two modes, which represent a single physical process, the EOF3 mode may by influenced by two independent physical modes of variability: changes in the DWBC and arrival of westward-propagating open-ocean eddies on the offshore edge of the GS. To demonstrate the DWBC's effects, the DWBC's volume transport is computed by integrating over the area of negative $\overline{{v }_{nor}}$ based on the coordinate system as described in section "Mean state of vertical profiles of western boundary currents near Cape Hatteras". Figure 4e shows the 30-day running mean of PC3 and the anomaly of DWBC volume transport. PC3 is correlated with the volume transport of the DWBC (r = 0.66). To explain the effects of the DWBC, we show the positive and negative composite mean vertical velocity fields in Fig. 5e,f, respectively, using the thresholds of the 80th percentile and 20th percentile. During the positive phase of PC3, the DWBC occupies the entire water column beneath the onshore edge of the GS, and the mean southward flow in its core region is − 0.2 m/s. In contrast, a much weaker DWBC only exists in water depths between 500 and 1500 m during the negative phase of PC3. Upper-ocean eddies' impacts are also captured by REOF mode 3. During the positive phase of PC3 (Fig. 5g), a cyclonic eddy on the southeast side of the transect influences the velocity field. An anti-cyclonic eddy in the same location during the negative phase of PC3 (Fig. 5h) accelerates the offshore velocity along the transect. Therefore, it is likely that the third mode is driven by both ocean eddies in the upper ocean and the DWBC in the deep ocean. It is possible that these two processes are causally linked, and that the arrival of a westward propagating eddy directly affects the DWBC by displacing the thermocline and thus influencing the thickness (and potential vorticity) of the layers comprising the DWBC. A similar interaction between the upper- and deep-ocean has been invoked for the Tail of the Grand Banks where the North Atlantic Current (the downstream extension of the Gulf Stream) and the DWBC interact over the complex topography of the Southeast Newfoundland Rise^42^. In that case, the position of the Gulf Stream relative to the topography sets whether Labrador Sea Water can pass in the ‘open valve’ state or is blocked in the ‘closed valve’ state^43^.

## Summary

A very high-resolution data-assimilative model is exploited to examine the western boundary current system off Cape Hatteras, North Carolina, USA, comprising the DWBC and the GS. During a cruise on the *R/V Neil Armstrong* in April 2017, five GS cross-shelf velocity sections were measured using shipboard ADCPs with hydrography measured by CTD casts and these are used here to ground-truth the model. The velocity fields and vertical density stratification simulated by the model are in good agreement with these in situ observations. This justifies using the model output to examine the crossover in more detail than can be achieved with in situ measures of the GS and DWBC. The modeling results confirm the persistence of the equatorward-flowing DWBC beneath the poleward-flowing GS near Cape Hatteras during the PEACH experiment period covered by the model run (January 2017 to December 2018).

The two-year averages of the modeled velocity fields and temperature profiles along five cross-stream transects near Cape Hatteras show that as the equatorward-flowing DWBC approaches Cape Hatteras, the thermocline depth deepens by 25 m over a 70 km distance due to the increase in ocean depth. Where the DWBC encounters the shoaling bathymetry from Cape Hatteras to the SAB, the thermocline depth increases by 10 m over a 73 km distance. This deepening or shallowing of thermocline depth is of the correct sign to maintain a constant layer thickness in the DWBC. However, the changes in bottom depth surpass the changes in the modeled thermocline depth by an order of magnitude so maintaining a constant layer thickness in the DWBC cannot entirely explain the observed variations in thermocline depth. Future studies will investigate other factors that might contribute to the modeled variations in thermocline depth and will explore the connections between the different components of the DWBC and the changes in ocean bathymetry.

Spatial variability is also examined in the modeled DWBC and GS mean volume transports. The transport of the DWBC decreases by a factor of about two over only 160 km, from 22.3 Sv at the northernmost transect to 10.6 Sv at the southernmost transect. The GS transport, on the other hand, remains relatively stable in the downstream direction. The GS transport increases by 11%, from 67.3 Sv at transect 1 to 75.2 Sv at transect 3, before fluctuating slightly from transect 1 to 5, with transport shifting from 73 to 74 Sv.

The Rotated Empirical Orthogonal Function (REOF) analysis is applied to examine the variability of the model’s western boundary currents at 36°N near Cape Hatteras. The first mode, GS meander (lateral position change), accounts for 55.3% of the velocity variance. The second mode, related to wind forcing, reflected by upwelling-favorable or downwelling-favorable wind conditions, explains 12.5% of the velocity variance. The third mode, driven by both the DWBC and upper-ocean eddies, accounts for 7.1% of the velocity variance. Both mode 1 and mode 2 are surface intensified features. In contrast, mode 3 exhibits a dipole characteristic: one within the upper 500-m water column and another associated with the DWBC in its deeper ocean. Future research could investigate whether there is a connection between the DWBC transport changes and the arrival of upper-ocean eddies from the ocean interior (i.e., does the arrival of eddies on the offshore edge of the GS/DWBC system serve as a “valve” that controls DWBC transport). The arrival of an offshore eddy may locally impact the flow of the DWBC near Cape Hatteras.

Our analysis based on a very high-resolution ocean reanalysis over two years highlights the impact of GS meandering, atmospheric forcing, the DWBC, and open-ocean eddies on the western boundary current velocity fields at 3°N near Cape Hatteras. Better understanding and prediction of ocean circulation variability in this region requires comprehensive understanding of these individual components and their interactions over longer time scales.

## Data Availability

Observational shipboard Acoustic Doppler Current Profiler dataset is available through Rolling Deck to Repository (R2R): https://doi.org/10.7284/125889. Ocean reanalysis data can be requested made to the corresponding author: Ruoying He at rhe@ncsu.edu.
